# Significant benefit of Nivolumab treating PD-L1 positive metastatic pulmonary carcinosarcoma: a case report and literature review

**DOI:** 10.18632/oncotarget.19089

**Published:** 2017-07-07

**Authors:** Zhe Zhang, Yishan Chen, Mingxia Ma, Junli Hao, Rui Ding, Lixin Han, Jiayun Zou, Lina Zhang, Qin Meng, Xiujuan Qu, Yunpeng Liu, Mingfang Zhao

**Affiliations:** ^1^ Department of Medical Oncology, The First Hospital of China Medical University, Shenyang, Liaoning, China; ^2^ Suzhou Junmeng Bioscience Co., Ltd, Suzhou City, Jiangsu Province, China

**Keywords:** pulmonary carcinosarcoma, programmed cell death protein-1 (PD-1), nivolumab, immunotherapy

## Abstract

Immunotherapy has recently become a new focus for the treatment of malignant tumors following the surgery, chemotherapy, radiotherapy, and molecular targeted therapy. Nivolumab, a human monoclonal antibody, is the first programmed cell death protein-1 (PD-1) inhibitor, which can prohibit the interaction of its ligand (PD-L1), restoring the immune response of T cells, and enhancing the recognition of tumor cells by the immune system. Pulmonary carcinosarcoma is an uncommon but highly aggressive tumor type with a poor prognosis. We described a case of pulmonary carcinosarcoma, with the positive expression of PD-L1, obtained a significant benefit from Nivolumab treatment in a 64-year-old Chinese man, which give us a clue that patients with pulmonary carcinosarcoma may benefit fromanti-PD-1 immunotherapy.

## INTRODUCTION

Pulmonary carcinosarcoma is a rare type of tumor with a characteristic of strong invasiveness, accounted for 0.1% of all malignant lung tumors [1]. It is defined as malignancy, composed of a mixture of carcinoma and sarcoma elements by the World Health Organization (WHO). This disease was characterized by rapid tumor growth, invasion, metastasis and recurrence [2] and low sensitivity to the chemotherapy treatment [3]. The programmed death-1 (PD-1) is expressed in cytotoxic T cells and T-regulatory cells activated in response to inflammation or infection in peripheral tissues [4]. The Bind of the programmed death ligand-1 (PD-L1) to PD-1 limits the T cells’ response to the stimuli, causing an immune suppression [5]. Cancer cells can induce the PD-1 expression, called immunosuppressive, allow itself to be “hidden” from natural immune attack [6]. Anti-PD-1 therapies disrupt this pathway and make activated cytotoxic T cells available to attack the cancer cells [6]. Nivolumab, a fully humanimmunoglobulin G4 (IgG4) monoclonal antibody, is the first PD-1 inhibitor that can obstruct the PD-1 interacting with its ligand [7]. Blocking PD-1 signaling cascade attenuate lymphocyte apoptosis, thereby restoring the immune response of T cells, and enhancing the recognition of tumor cells by the immune system [8]. Nivolumab has been approved to treat melanoma and metastatic nonsmall-cell lung cancer (NSCLC) by Food and Drug Administration (FDA) of the United States of American [9, 10]. Nivolumab, as the representative of immunotherapy, brings hope for the treatment of aggressive and evasive tumors. Here, we first reported a case of a 64-year-old Chinese man, diagnosed as pulmonary carcinosarcoma, with the positive expression of PD-L1, obtained a significant benefit from Nivolumab treatment.

## CASE PRESENTATION

### Basic information

A 64-year-old, Chinese male, had a 10-year history of type 2 diabetes, using insulin to control blood glucose. He never smoked or consumed alcohol before. His mother and sister both died of lung cancer.

### Treatment

**Figure 1:**
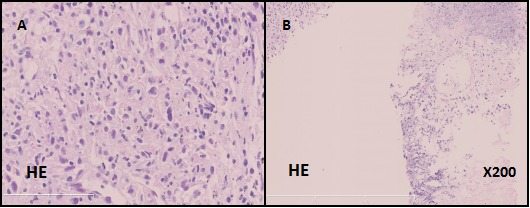
Microscopic findings of the patient's lung biopsy specimen Poorly differentiated sarcomatoid-like spindle cancer cells. (**A**, hematoxylin and eosin, 400X). Both the lung tissue and cancer cells (**B**, hematoxylin and eosin, 200X)

The patient went to Beijing Tongren Hospital on July 23, 2015, because of the lasting abdominal pain for more than 1 month. Abdominal computed tomography (CT) revealed that the right adrenal gland accounted for the possibility of hemorrhage lesions with rupture. Pulmonary CT scan showed that left lung apicoposterior segment accounted for lesions, controlateral pulmonary metastasis, left hilar and mediastinal lymph node metastasis and right pleural effusion. The pathologic stage of disease was IV ( T4N3M1) according to Eighth edition of lung cancer TNM staging from Union for International Cancer Control (UICC). On August 20, 2015, patient underwent percutaneaous left lung needle biopsy and the pathological analysis identified: (1) Malignant pleural mesothelioma or synovial sarcoma; (2) metastatic adrenal cortical carcinoma or renal cell carcinoma. Immunohistochemistry results showed: CD20 (-), LCA (-), CD3 (-), vimentin (+), CK-PAN (+), S-100 (-). After the consultation with the Cancer Hospital of Chinese Academy of Medical, under the microscope can we see poorly differentiated sarcomatoid-like spindle cancer cells (Figure 1), so it was finally diagnosed as pulmonary carcinosarcoma. The full-genome scan (Next Generation Sequencing, NGS) of this patient suggested that EGFR, KRAS, BRAF and ALK, the commonest driving genes of lung cancer, were all identified as wild type. Besides, genes including HER2, PI3K, MET, which is relevant for pulmonary sarcomatoid carcinoma, have no mutations. However, we found polymorphismof the gene of NAD(P)H quinone oxidoreductase 1 (NQO1) in P187S(rs1800566).

**Figure 2:**
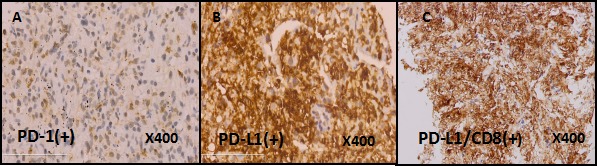
The IHC staining of PD-1 , PDL-1 and PDL-1/CD8 in the patient's lung biopsy specimen The expression of PD-1 is positive (**A**, Immunohistochemical staining, clone: SP269, Spring 195 Bioscience, Inc., Pleasanton, CA, USA). The expression of PD-L1 is positive (**B**, Immunohistochemical staining, clone: SP142, Spring 195 Bioscience, Inc., Pleasanton, CA, USA). The PD-L1/CD8 double staining is positive (**C**, Immunohistochemical staining, clone: SP239, Spring 195 Bioscience, Inc., Pleasanton, CA, USA).

Positron emission tomography-computed tomography (PET-CT) on September 1, 2015 showed that, in line with the left lung cancer, it was associated with contralateral pulmonary, left lung lymph node, bilateral adrenal metastasis and a little obstructive inflammation in left lung lobe, there was no significant increase uptake in mediastinal lymph node. Immunohistochemistry staining of lung biopsy specimen showed positive for PD-L1 and PD1 (Figure 2). PD-L1/CD8 double staining showed that over 10% tumor cells hada strong positive expression of PD-L1. In addition, there had CD8+ T cells invasion within tumor cells (Figure 2). Because of the large tumor load and the poor physical fitness, the patient refused to use chemotherapy.

**Figure 3:**
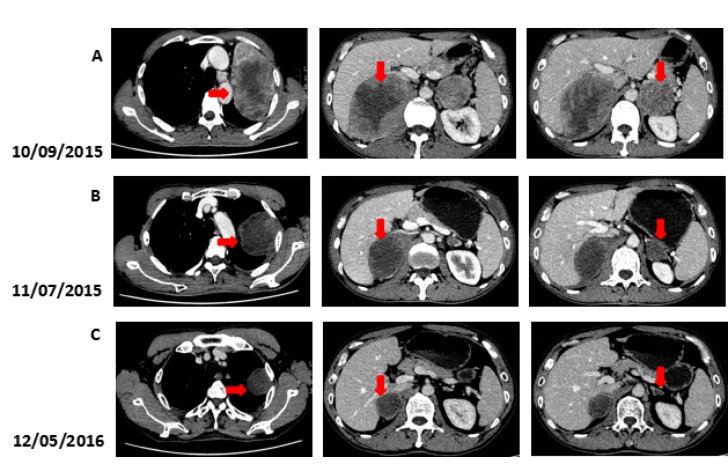
Computed tomography (CT) findings (**A**) The CT scan shows the lesion in left lung and bilateral adrenal (arrows). (**B**, **C**) A restaging CT scan shows the lesion in left lung and bilateral adrenal keep shrinking after two, fifteen courses of Nivolumab (arrows).

**Figure 4:**
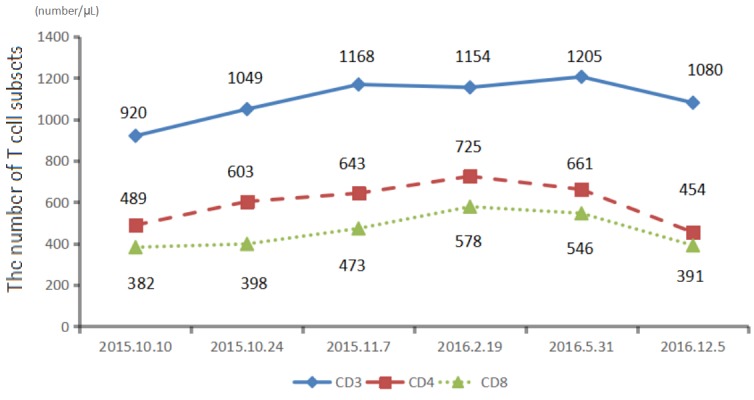
The number of T cell subsets of patien's peripheral blood increased to normal after the treatmen

**Figure 5:**
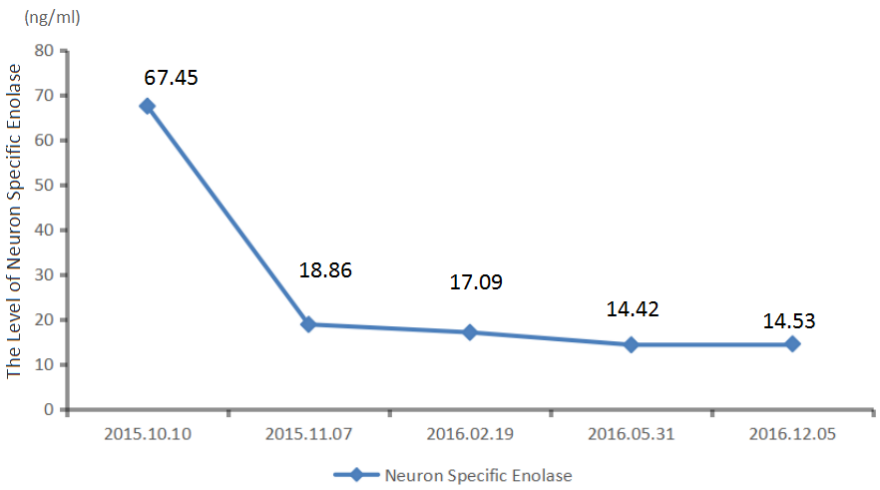
The level of neuron specific enolase of patien's peripheral blood keeps falling during treatment

**Figure 6:**
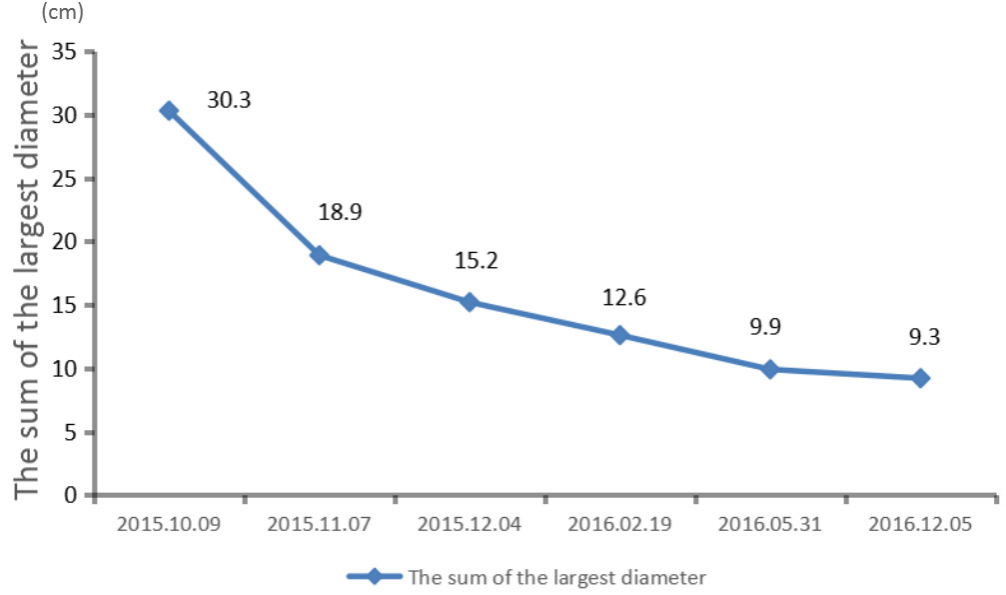
The lesion keeps shrinking during treatment

We recommended immunotherapy with Nivolumab at a dose of 3 mg/kg, once every 2 weeks since October 10, 2015. The first three days after the administration, the patient had fever, and the maximal body temperature of 39.2°C. After symptomatic treatment of fever, patient's body temperature returned to normal. The next day, the patient felt left anterior chest relaxation. A week later, the chronic back pain was significantly reduced, and overall physical fitness was improved although there was still a mild cough. Occasionally bloody sputum and dyspnea symptoms disappeared completely. The results of the two periodic imaging showed that the left lung and bilateral adrenal lesions were dramatically decreased. The evaluation of response rate is partial remission (PR) based on Recist1.1 standard. Subsequently, the patient continues to receive 5 cycles, 10 cycles, 15 cycles and 24 cycles of immunotherapy with Nivolumab. Because of the good effect, the patient uses Nivolumab every 3 weeks since the 21^th^ cycles. The imaging results indicated that the lesion was continuously to shrink (Figure 3, Figure 6). The number of T cell subsets of patient's peripheral blood increased to normal after the above-mentioned therapy (Figure 4). Only neuron specific enolase (NSE) is abnormal among all tumor markers in this patient, but it was decreased to normal range after the following treatment (Figure 5). The progress-free survival (PFS) time of this patient has not been reached.

## DISCUSSION

Pulmonary carcinosarcoma is rarely diagnosed in our clinical work, with poor prognosis, and high incidence of distant metastasis [11]. Early discovery and radical resection is the best therapeutic choice [12, 13]. Metastatic pulmonary carcinosarcoma is especially not sensitive to radiotherapy and chemotherapy, the median survival time is short [12, 13].

Lung cancer has long been considered as poorly immunogenic [14]. However, with the deeper understanding of the immunotherapy, it was found that tumor microenvironment can protect tumor cells against the recognition and damage of the immune system [15]. The immune escape mechanism plays an important role in the occurrence and development of tumors [16]. The PD-1 (also known as CD279) is expressed on activated T and B cells in response to inflammation or infection in peripheral tissues, which can dampen the immune response [4, 5, 17]. PD-1 is engaged by ligands PD-L1 (B7-H1, CD274) and PD-L2 (B7-DC, CD273), expressed by tumor cells and infiltrating immune cells [18, 19]. Binding of the PD-L1 to PD-1 inactivates the T cell to limit the immune response to the stimuli, which causes an immune suppression [4, 5]. Cancer cells enhance the immunosuppressive action of this pathway by inducing PD-1 expression, which ultimately allow the cancer to be “hidden” from natural immune attack [6]. Anti-PD-1 therapies disrupt this pathway by preventing the PD-1 ligand from binding to its receptor, leaving activated cytotoxic T cells available to attack the cancer cells [16]. The PD-1/PD-L1 signaling pathway has been widely recognized and valued in clinical tumor immunotherapy, which has brought a new direction and insight for tumor immunotherapy [20–23].

The anti-PD-1 antibody is one of immune checkpoint inhibitors, which has been added to many treatments against different types of cancers, including lung cancer [24]. Nivolumab (BMS-936558, or MDX1106, trade name Opdivo; Bristol-Myers Squibb, Princeton, NJ, USA) is a fully human IgG4 monoclonal antibody, which binds to PD-1 and blocks the interaction with PD-L1 and PD-L2, therefore, it prevents T-cell inhibition and restores antitumour immune responses [7, 24]. Nivolumab was approved by the US Food and Drug Administration (FDA) in 2015 for metastatic NSCLC, which is a new milestone in the history of the development of treatment for NSCLC [25]. In this report, we found that pulmonary carcinosarcoma, a non-adaptive disease, obtained a good clinical efficacy based on high expression of PD-L1 and PD-1, offering us an important clue for follow-up of patients with non-adaptive treatment, but also provide some reference for subsequent clinical basket studies.

As far as we know, it is the first case of a patient with pulmonary carcinosarcoma who was treated with Nivolumab after showing positive expression of PD-1 and PD-L1 by IHC (Immunological Histological Chemistry) test. After treatment, there was only a side effect of drug related fever, which lasted about 4 days, and then gradually improved. Tumor were rapidly decreased and obtained partial remission (PR) after two cycles of Nivolumab, and the lesions present a good trend to decrease in the follow-up treatment.It was confirmed that the anti PD-1 therapy has continued to shrink the tumor.

The good response and tolerance to the Nivolumabis consistent with the results of large clinical studies. In open-label, randomized, international, phase 3 trial, like Brahmer et al , there were 272 patients who received nivolumab intravenously as monotherapy (3 mg/kg every 2 weeks) or docetaxel (75 mg/m2 every 3 weeks) [26]. The median overall survival (OS) time was 9.2 months with nivolumab *versus* 6.0 months with docetaxel, the median PFS time of the nivolumab group was 3.5 months *versus* 2.8 months of the docetaxel group [26, 27]. The most frequently treatment-related adverse events in the nivolumab group were fatigue (in 16% of the patients), decreased appetite (in 11%), and asthenia (in 10%). Besides, 58% of the patients had any grade events, 7% had grade 3 or 4 events, and none had events of grade 5 [26].

The anti-PD-1/PD-L1 immune therapy is for removing inhibition of T cell. Our patient was treated with Nivolumab.After that, the number of T cells increased, confirmed that anti-PD-1 treatment help T cells restore the immune response [28]. In this case, PD-L1 was highly expressed in tumor tissues, and the invasion of CD8+ T cells to tumor cells was also discovered. Studies have reported that there is a positive correlation between the invasion of CD8+ T cells to tumor cells and the expression of the PD-L1 in immune cells, which confirms the adaptive immune escape mode, and indicates a poor survival benefit [29].

In addition, the expression of PD-1 in tumor cells is associated with the good clinical efficacy. Our patient had PD-1 expression in tumor cells, which is not only evidence of good therapeutic potential, but also provides reference for subsequent validation of the relationship between immunotherapy and the PD-1 expression in tumor cells [30].

The full-genome scan of this patient suggested that NQO1 gene has polymorphism in P187S. NQO1 gene P187S polymorphism (C609T mutation) can destroy the stability of the enzymecausing loss of activity, so the function of benzene poisoning protection is destructive, the polymorphism can increase the risk of hematotoxicity and leukemia in breast cancer, non-smoking lung cancer and people exposed to benzene [31–34]. This polymorphism of NQO1 was reported to be associated with decreased risk of lung cancer for women, especially with light smoking women [35]. However, in other studies, the polymorphism of NQO1 is related to the high risk of lung cancer [36]. As a result, the relationship between the polymorphism of NQO1 with the cause of this patient's disease is unclear. Besides, whether this has relationship with the expression of PD-1 and PD-L1 needed to be further confirmed.

## SUMMARY

In general, represented by Nivolumab, anti PD-1/PD-L1 immune therapy has been a new therapeutic approach for the treatment of solid tumors, and hopefully, immunotherapy can ameliorate the quality of patients’ life and lengthen the survival time of patients. In our case, the patient with pulmonary carcinasarcoma achieves a good therapeutic effect from the anti PD-1/PD-L1 immune therapy. However, we need more scientific and clinical research to provide more information about the anti PD-1/PD-L1 immune therapy toward the pulmonary carcinosarcoma.

## References

[1] Chuang TL, Lai CL, Chang SM, Wang YF (2012). Pulmonary carcinosarcoma: 18F-FDG PET/CT imaging. Clin Nucl Med.

[2] Olobatoke AO, David D, Hafeez W, Van T Saleh HA (2010). Pulmonary carcinosarcoma initially presenting as invasive aspergillosis: a case report of previously unreported combination. Diagn Pathol.

[3] Weissferdt A, Moran CA (2011). Malignant biphasic tumors of the lungs. Adv Anat Pathol.

[4] Topalian SL, Hodi FS, Brahmer JR, Gettinger SN, Smith DC, McDermott DF, Powderly JD, Carvajal RD, Sosman JA, Atkins MB, Leming PD, Spigel DR, Antonia SJ (2012). Safety, activity, and immune correlates of anti-PD-1 antibody in cancer. N Engl J Med.

[5] Pardoll DM (2012). The blockade of immune checkpoints in cancer immunotherapy. Nat Rev Cancer.

[6] Khalil DN, Smith EL, Brentjens RJ, Wolchok JD (2016). The future of cancer treatment: immunomodulation, CARs and combination immunotherapy. Nat Rev Clin Oncol.

[7] Guo L, Zhang H, Chen B (2017). Nivolumab as Programmed Death-1 (PD-1) Inhibitor for Targeted Immunotherapy in Tumor. J Cancer.

[8] Rizvi NA, Mazières J, Planchard D, Stinchcombe TE, Dy GK, Antonia SJ, Horn L, Lena H, Minenza E, Mennecier B, Otterson GA, Campos LT, Gandara DR (2015). Activity and safety of nivolumab, an anti-PD-1 immune checkpoint inhibitor, for patients with advanced, refractory squamous non-small-cell lung cancer (CheckMate 063): a phase 2, single-arm trial. Lancet Oncol.

[9] El-Osta H, Shahid K, Mills GM, Peddi P (2016). Immune checkpoint inhibitors: the new frontier in non-small-cell lung cancer treatment. Onco Targets Ther.

[10] Pilotto S, Kinspergher S, Peretti U, Calio A, Carbognin L, Ferrara R, Brunelli M, Chilosi M, Tortora G, Bria E (2015). Immune checkpoint inhibitors for non-small-cell lung cancer: does that represent a ‘new frontier’?. Anticancer Agents Med Chem.

[11] Digesu CS, Wiesel O, Vaporciyan AA, Colson YL (2016). Management of Sarcoma Metastases to the Lung. Surg Oncol Clin N Am.

[12] Mori S, Uehara H, Motoi N, Okumura S (2017). Pulmonary artery sarcoma presenting as an isolated lung mass. Gen Thorac Cardiovasc Surg.

[13] Varghese M, Bruland O, Wiedswang AM, Lobmaier I, Røsok B, Benjamin RS, Hall KS (2016). Metastatic mesenteric dedifferentiated leiomyosarcoma: a case report and a review of literature. Clin Sarcoma Res.

[14] de Mello RA, Pousa I, Pereira D (2015). Nivolumab for advanced squamous cell lung cancer: what are the next steps?. Lancet Oncol.

[15] Sundar R, Cho BC, Brahmer JR, Soo RA (2015). Nivolumab in NSCLC: latest evidence and clinical potential. Ther Adv Med Oncol.

[16] Mitchell PL, John T (2016). Lung cancer in 2016: immunotherapy comes of age. Lancet Respir Med.

[17] Hamid O, Robert C, Daud A, Hodi FS, Hwu WJ, Kefford R, Wolchok JD, Hersey P, Joseph RW, Weber JS, Dronca R, Gangadhar TC, Patnaik A (2013). Safety and tumor responses with lambrolizumab (anti-PD-1) in melanoma. N Engl J Med.

[18] Belum VR, Benhuri B, Postow MA, Hellmann MD, Lesokhin AM, Segal NH, Motzer RJ, Wu S, Busam KJ, Wolchok JD, Lacouture ME (2016). Characterisation and management of dermatologic adverse events to agents targeting the PD-1 receptor. Eur J Cancer.

[19] Topalian SL, Sznol M, McDermott DF, Kluger HM, Carvajal RD, Sharfman WH, Brahmer JR, Lawrence DP, Atkins MB, Powderly JD, Leming PD, Lipson EJ, Puzanov I (2014). Survival, durable tumor remission, and long-term safety in patients with advanced melanoma receiving nivolumab. J Clin Oncol.

[20] Sznol M (2014). Blockade of the B7-H1/PD-1 pathway as a basis for combination anticancer therapy. Cancer J.

[21] Sznol M, Chen L (2013). Antagonist antibodies to PD-1 and B7-H1 (PD-L1) in the treatment of advanced human cancer. Clin Cancer Res.

[22] Zou W, Wolchok JD, Chen L (2016). PD-L1 (B7-H1) and PD-1 pathway blockade for cancer therapy: Mechanisms, response biomarkers, and combinations. Sci Transl Med.

[23] Adachi K, Tamada K (2015). Immune checkpoint blockade opens an avenue of cancer immunotherapy with a potent clinical efficacy. Cancer Sci.

[24] Li Y, Li F, Jiang F, Lv X, Zhang R, Lu A, Zhang G (2016). A Mini-Review for Cancer Immunotherapy: Molecular Understanding of PD-1/PD-L1 Pathway & Translational Blockade of Immune Checkpoints. Int J Mol Sci.

[25] Kazandjian D, Suzman DL, Blumenthal G, Mushti S, He K, Libeg M, Keegan P, Pazdur R (2016). FDA Approval Summary: Nivolumab for the Treatment of Metastatic Non-Small Cell Lung Cancer With Progression On or After Platinum-Based Chemotherapy. Oncologist.

[26] Brahmer J, Reckamp KL, Baas P, Crinò L, Eberhardt WE, Poddubskaya E, Antonia S, Pluzanski A, Vokes EE, Holgado E, Waterhouse D, Ready N, Gainor J (2015). Nivolumab versus Docetaxel in Advanced Squamous-Cell Non-Small-Cell Lung Cancer. N Engl J Med.

[27] Yaqub F (2015). Nivolumab for squamous-cell non-small-cell lung cancer. Lancet Oncol.

[28] Thompson ED, Zahurak M, Murphy A, Cornish T, Cuka N, Abdelfatah E, Yang S, Duncan M, Ahuja N, Taube JM, Anders RA, Kelly RJ (2017). Patterns of PD-L1 expression and CD8 T cell infiltration in gastric adenocarcinomas and associated immune stroma. Gut.

[29] Brahmer JR, Hammers H, Lipson EJ (2015). Nivolumab: targeting PD-1 to bolster antitumor immunity. Future Oncol.

[30] Kleffel S, Posch C, Barthel SR, Mueller H, Schlapbach C, Guenova E, Elco CP, Lee N, Juneja VR, Zhan Q, Lian CG, Thomi R, Hoetzenecker W (2015). Melanoma Cell-Intrinsic PD-1 Receptor Functions Promote Tumor Growth. Cell.

[31] Guo S, Gao M, Li X, Li Y, Chu S, Zhu D, Niu W (2012). Lack of association between NADPH quinone oxidoreductase 1 (NQO1) gene C609T polymorphism and lung cancer: a case-control study and a meta-analysis. PLoS One.

[32] Liu Y, Zhang D (2011). The NQO1 C609T polymorphism and risk of lung cancer: a meta-analysis. Asian Pac J Cancer Prev.

[33] Lou Y, Li R, Xiong L, Gu A, Shi C, Chu T, Zhang X, Gu P, Zhong H, Wen S, Han B (2013). NAD(P)H: quinone oxidoreductase 1 (NQO1) C609T polymorphism and lung cancer risk: a meta-analysis. Tumour Biol.

[34] Lajin B, Alachkar A (2013). The NQO1 polymorphism C609T (Pro187Ser) and cancer susceptibility: a comprehensive meta-analysis. Br J Cancer.

[35] Timofeeva M, Kropp S, Sauter W, Beckmann L, Rosenberger A, Illig T, Jäger B, Mittelstrass K, Dienemann H, Bartsch H, Bickeböller H, Chang-Claude J, Risch A, Wichmann HE, LUCY-Consortium (2010). Genetic polymorphisms of MPO, GSTT1, GSTM1, GSTP1, EPHX1 and NQO1 as risk factors of early-onset lung cancer. Int J Cancer.

[36] Liang GY, Pu YP, Yin LH (2004). [Studies of the genes related to lung cancer susceptibility in Nanjing Han population, China]. [Article in Chinese]. Yi Chuan.

